# Enhanced carbon utilization and storage: An application of nonionic-based binary surfactant CO_2_ foam

**DOI:** 10.1016/j.heliyon.2025.e42561

**Published:** 2025-02-11

**Authors:** Ayomikun Bello, Anastasia Ivanova, Denis Bakulin, Kirill Maerle, Denis Davydov, Artem Penigin, Alexey Cheremisin

**Affiliations:** a Center Petroleum Science and Engineering, Skolkovo Institute of Science and Technology, Skolkovo Innovation Center, 11 Sikorskiy Street, Moscow 143026, Russia; b «GK Gazpromneft», Saint Petersburg, Russia

**Keywords:** CO_2_ storage, EOR, Carbonate, High salinity, Depleted

## Abstract

Mobility control of CO_2_ is important for effective subsurface utilization and sequestration of anthropogenic CO_2_ in depleted formations. This not only enhances oil recovery but also increases CO_2_ storage efficiency, addressing a key challenge for a future zero-carbon economy. In this study, novel techniques were developed by injecting CO_2_ foam generated with a nonionic-based binary surfactant system to improve geological CO_2_ storage and to co-optimize carbon utilization and storage efficiency in high salinity carbonate porous media, based on hypotheses from our previous works. Carbonate core samples from a producing oil field were used as the porous media to evaluate oil production and CO_2_ storage with varying injectants. The introduction of foam as a mobility control fluid significantly increased the CO_2_ retention factor by 4.6 times. With the addition of a nonionic-based binary surfactant system as a foaming agent, the retention factor further increased to 5.6 times. This is in addition to an 85% increase in oil recovery. This study demonstrates the potential of surfactant foams to control the mobility of injected gas, thereby enhancing CO_2_ retention in carbonate porous media. The findings highlight the effectiveness of nonionic-based binary surfactant systems as foaming agents for the co-optimization of carbon utilization and storage in depleted oil formations.

## Graphical abstract

**Figure:**
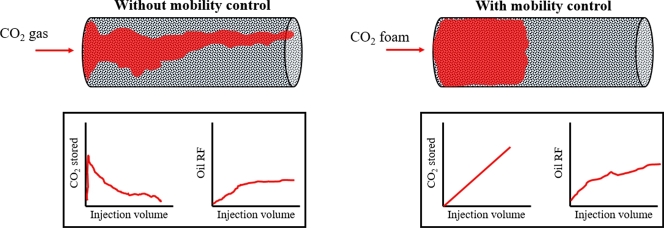

## 1 Introduction

Anthropogenic CO_2_ emission is a significant concern due to its role in global warming and climate change. This is particularly relevant in the oil industry, where the production process contributes to these emissions [1], [2]. Conventional oil production methods are energy intensive and contribute substantially to greenhouse gas emissions. Therefore, a more efficient technique of oil production with less CO_2_ production is crucial [3], [4].

In oil production, the injection of gases such as CO_2_, N_2_, and CH_4_, stands out as an important technique for enhancing and maximizing oil production from reservoirs. Among these gases, CO_2_ is more commonly used, due to its properties, compatibility with various reservoir types, and its efficiency in stimulating hydrocarbon flow [5], [6]. Its application is particularly advantageous in mature or depleted reservoirs where primary and secondary recovery methods have become less efficient.

One of the primary mechanisms through which CO_2_ facilitates enhanced oil recovery is its ability to lower the viscosity of crude oil. As CO_2_ dissolves in the oil phase, it reduces the interfacial tension between oil and water, thereby enabling the oil to flow more readily through the reservoir rock pores [7], [8]. Additionally, the miscibility of CO_2_ with hydrocarbons enables it to act as a solvent, effectively solubilizing and mobilizing trapped oil, which further facilitates its displacement towards production wells. Moreover, the injection of CO_2_ can lead to reservoir pressure maintenance, a critical factor in sustaining production rates over the long term [8], [9]. As natural reservoir pressure declines with ongoing extraction, CO_2_ injection serves to replenish and uphold reservoir pressure levels, thereby extending the productive lifespan of the reservoir.

One pivotal aspect in enhancing the efficiency of CO_2_-EOR lies in maximizing CO_2_ retention within the reservoir [10], [11]. However, while CO_2_-EOR can significantly enhance oil recovery, it also presents several challenges. The injected CO_2_ can break through to production wells, leading to increased expenses for separation and potential emissions of CO_2_ to the atmosphere [12], [13], [8], [10]. Thus, conventional methods of CO_2_ injection do not often achieve optimal CO_2_ storage due to various factors such as buoyancy effects, gravitational segregation, and inefficient sweep efficiency.

To address these challenges and enhance CO_2_ storage capacity, there is a growing interest in using CO_2_ foams as alternative injection fluids in combined EOR and sequestration operations [14], [15], [16], [10]. CO_2_ foam is a dispersion of gas bubbles in a liquid phase stabilized by surfactants which exhibits distinct rheological and interfacial properties that offer advantages over conventional CO_2_ flooding methods. By generating a stable foam phase, the mobility of CO_2_ within the reservoir can be significantly reduced, thereby promoting better conformance and improving sweep efficiency [10], [17], [18]. This not only enhances the volumetric sweep efficiency but also mitigates the risk of early breakthrough. Moreover, the viscoelastic properties of CO_2_ foams enable them to effectively displace oil from reservoir pores and fractures, enhancing the recovery factor compared to conventional CO_2_ flooding techniques. Furthermore, CO_2_ foams create a larger interfacial area with the reservoir rock, which facilitates increased mass transfer and capillary trapping of CO_2_ [15], [19]. This is particularly advantageous in heterogeneous reservoirs where the gas during CO_2_ injection may bypass significant portions of the oil-bearing formation.

Carpenter [20] and Alcorn et al. [14] both highlighted the potential of in-situ generated foam to enhance CO_2_ storage and improve sweep efficiency during EOR. Zhang et al. [21] further emphasizes the role of foam-assisted CO_2_ EOR in enhancing oil recovery, with a focus on tight oil reservoirs. In Qi et al. [22], CO_2_ and brine was injected into the aquifer of a depleted oil formation. Their findings showed that a high percentage, about 80% - 95% of CO_2_ was rendered immobile in pore-scale droplets within the porous rock. Thus, injecting a water phase together with gas can lead to improved storage of CO_2_ as a trapped phase. In Yu et al. [23], the authors evaluated the use of CO_2_ foam for EOR and CCUS, with the aim of optimizing foam performance to improve CO_2_ storage potential in sandstone and carbonate core sample. Their results showed that the sandstone core showed a higher storage potential than the carbonate core sample. The authors asserted that injecting CO_2_ as foam improved the injectant mobility and increased sweep efficiency. These results were also supported in Lyu et al. [24], where it was seen that foams reduced gas mobility effectively by trapping gas bubbles and inhibiting CO_2_ from migrating upward in the presence of gravity.

As highlighted in Føyen et al. [25], foam generation behavior and foam strength are essential for enhancing sweep efficiency and increasing CO_2_ storage capacity during sequestration of anthropogenic CO_2_. In their study, the authors evaluated six surfactants for generating CO_2_-foam at reservoir conditions and their results showed that CO_2_ storage capacity increased by around 27%. Similarly, Adebayo [26], showed that foams can plug a significant fraction of the pores in a porous medium, which can subsequently cause a significant trapping of the injected gas. Therefore, the ability of the foam to plug rock pores to trap CO_2_ is a function of the foam strength and the capillary pressures in the rock.

The potential of CO_2_ foam for CO_2_ utilization and storage is an area of growing interest. Surfactants are the main components in a foam system, facilitating foam generation by reducing the gas-water interfacial tension and stabilizing the thin films between bubbles. However, previous studies have identified several key challenges related to surfactants, including high rock adsorption, which causes poor foamability and stability [27], [28], [29], [30]. Adsorption leads to the loss of valuable chemical components from the solution, significantly reducing surfactant concentration in chemical slugs. Consequently, a substantial portion of injected surfactant molecules is lost to porous media due to adsorption, leaving insufficient concentration for efficient foam generation. This often necessitates the injection of large volumes of surfactant solutions, increasing operational expenses [29].

Furthermore, foams stabilized with conventional commercial surfactants typically cannot maintain their stability under harsh reservoir conditions due to their high propensity to degrade under high temperature and salinity [31], [10], [15]. Therefore, this work studies the application of a nonionic-based binary surfactant system as an alternative to the conventional commercial foaming agents in core flooding experiments for CO_2_ utilization and storage in high salinity carbonate core samples.

Building on our previous studies, which developed, screened, and characterized a wide range of binary surfactant systems [32], we selected the best compositions to investigate their ability to mitigate rock adsorption [33] and maintain foam stability in the presence of oil [34]. These binary systems have demonstrated improved foam stability and reduced rock adsorption compared to single surfactants.

In this study, we integrated the conventional core flooding setup with a gas chromatography unit at the outlet to monitor the dynamics and composition of the produced gas in real-time. By analyzing the composition of the produced gas, we precisely quantified the volume of CO_2_ produced at every pore volume injected, a critical metric for studies related to carbon utilization and storage. Besides the baseline experiment of CO_2_ injection, we compared the carbon utilization and storage potentials of two sets of foaming compositions: CO_2_ foam generated with conventional single surfactants and CO_2_ foam generated with a binary surfactant system comprising a nonionic surfactant and a zwitterionic surfactant. This study is significant for its potential to simultaneously reduce greenhouse gas emissions and enhance oil production, presenting a sustainable technique for the petroleum industry. The findings of this work are particularly valuable in the context of the Intergovernmental Panel on Climate Change (IPCC)'s decarbonization strategy, which aims to limit global warming to between 1.5 and 2 °C [35].

## 2 Materials and methodology

### 2.1 Materials

This work was conducted for a carbonate oil field characterized with high salinity. Hence, all the experimental materials were obtained from the carbonate field, and the conditions of the experiments were designed to replicate the reservoir conditions.

*Formation water:* The reservoir brine was used to establish the irreducible water saturation of the core plugs. The composition is detailed in Table 1.

**Table 1:** Brine composition.

Salt	NaCl	KCl	CaCl_2_	MgCl_2_
Composition, g/L	172.056	4.261	42.172	15.7

*Oil:* A live crude oil model was used in this study. The dead oil, initially obtained from the field, was recombined with natural gas to obtain the live oil model. The final properties of the oil model used in the experiments are presented in Table 2. The crude oil was primarily composed of saturates (67%) and asphaltenes (18%), with the remainder being aromatics and resins.

**Table 2:** Characteristics of the reservoir oil models used in this study.

Density (*g*/*cm*^3^)	Viscosity (*mPa.s*)	Gas factor
0.842	3.13	73

*Gas:* CO_2_, with a purity of 99.99%, was purchased from Moscow Gas Processing Plant. In this study, it was used as a displacing agent for enhanced oil recovery and for foam generation.

*Surfactants:* Two surfactant systems were used in this study for foam generation, namely BETAINE and BETFAR. The individual surfactants comprised Cocamidopropyl Betaine (BETAINE) - a zwitterionic surfactant. The binary surfactant systems were combinations of zwitterionic (BETAINE) and nonionic (FARUS), mixed in a 1:2 ratio, following previous preliminary results in Bello et al. [32]. FARUS is an oxyethylated phenol in monoatomic alcohol (FARUS) - a nonionic surfactant. The surfactants used for foam generation in this study were selected based on previous screening tests and results from our previous foam stability and surfactant adsorption studies [34], [33]. All surfactants were used as received, and all studies were performed under reservoir conditions of 41 °C and 21 MPa. Further details of the surfactant systems are given in Table 3.

**Table 3:** Surfactant compositions and their concentrations.

Surfactant composition	Active content	Concentration, wt.%
FARUS - Oxyethylated phenols in monoatomic alcohols	50%	0.480%
BETAINE - Cocamidopropyl betaine	75%	0.525%
BETFAR - BETAINE + FARUS (1:2)	-	0.120%

*Core samples:* Carbonate core samples were obtained from the oil field mentioned earlier, and they consist of calcite (83.5%), Dolomite (9.5%) and Illite (7%). Their petrophysical properties are shown in Table 4.

**Table 4:** Petrophysical properties of core samples used in this study.

Model tag	Length (cm)	Diameter (cm)	Porosity (%)	Permeability (mD)	Irreducible water saturation (%)	Displacing fluid
No. 1	6	3	8.18	0.0883	8.26	CO_2_ gas
No. 2	6	3	8.87	0.0582	39.18	CO_2_ foam (single surfactant)
No. 3	6	3	9.99	0.0786	13.87	CO_2_ foam (binary surfactant)

### 2.2 Methodology

The detailed descriptions of the experimental procedures followed in this study are given below.

#### 2.2.1 Preparation of core samples

In this study, we chose three models of porous media comprising of 2 core samples each from the same carbonate reservoir formation. The core samples were initially cleaned for 3 weeks with a 1:1 solvent mixture of methanol and toluene through a reflux extraction using a Soxhlet apparatus. Following this, the core samples were dried in a vacuum oven at 60 °C for about seven days.

#### 2.2.2 Core flooding set-up and injection procedures

**Figure 1:**
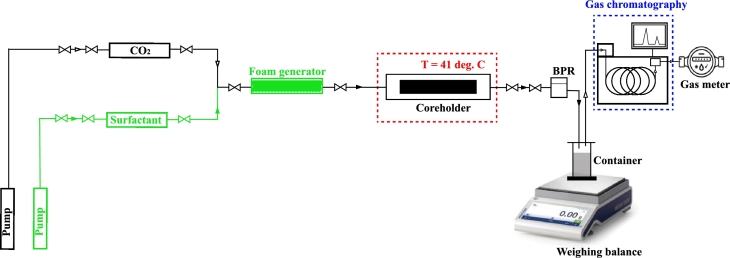
Experimental setup.

The core flooding experiments consisted of one CO_2_ flooding experiment and two CO_2_ foam flooding experiments, i.e. CO_2_ foam generated with single surfactant and binary surfactant systems. After vacuuming the cores for approximately 12 hours, they were saturated with the brine earlier described in Table 1. The cores were then centrifuged to achieve irreducible water saturation. The experimental setup, illustrated in Fig. 1, involved loading the core samples into a horizontally positioned hassler core holder.

Fluids were injected using plunger pumps *(GEOLOGIKA, Novosibirsk, Russia)*, beginning with hexane to establish thermobaric conditions within the core samples. Initially, hexane was injected to saturate the core samples to establish thermobaric conditions. After stabilizing these conditions for about 12 hours, crude oil was injected to displace the hexane and saturate the core samples. Before injecting CO_2_ or foam for oil recovery and CO_2_ storage assessments, the core samples contained known saturations of crude oil and irreducible water. All experiments were conducted at 41 °C. Pressure and differential pressure measurements were taken using one pressure transducer *(METRAN, Moscow, Russia)* at the inlet and one differential pressure transducer *(METRAN, Moscow, Russia)* in the middle of the core holder.

During the displacement stage for CO_2_ flooding, CO_2_ was injected at a constant rate of 2.4 cm^3^/hr until no oil droplets were observed at the outlet. For foam injection, pregenerated foam was injected at reservoir conditions using surfactant formulations screened from previous experiments. Each foam slug consisted of a 25% (v/v) surfactant solution and a 75% (v/v) CO_2_ slug, achieving a foam quality of 75%. The co-injection of both phases was controlled with flow rates of 0.6 cm^3^/hr for the surfactant solution and 1.8 cm^3^/hr for CO_2_, and then injected into an in-house foam generator, which is a hollow tube filled with glass beads of sizes ranging from 200 to 300 microns. The inlet and outlets of the foam generator were equipped with filters of 140 and 90 microns, respectively. The inlet filter aids in the appropriate mixing of the two phases going into the foam generator, while the outlet filter prevents the glass beads from flowing out of the system. The crude oil used was from the target field, same as the core samples and formation water, and had been investigated previously in our previous study to ensure compatibility and stability with the foam, preventing significant destructive effects on the generated foams. The process was stopped when oil production at the outlet ceased.

In this study, the co-injection method was used for foam generation, allowing foam to be generated under reservoir conditions at the surface before injection into the core samples. While co-injection of CO_2_ and surfactant solution presents no injectivity issues in the laboratory, it may not be feasible at the field scale, where surfactant alternating gas (SAG) is a more viable approach.

In the experimental setup in Fig. 1, the green pathway is only used during foam injection and is ignored during pure CO_2_ injection. Furthermore, at the core holder's outlet, a gas chromatography unit *(Agilent Technologies, Santa Clara, USA)* and gas meter *(Ritter, Gröbenzell, Germany)* were connected to analyze the CO_2_ volume in the produced gas effectively.

#### 2.2.3 Determination of assessment parameters

The experiments conducted in this study primarily investigate the efficiency of CO_2_ foams for carbon utilization and storage. Furthermore, we evaluated the feasibility of CO_2_ foam generated with a binary surfactant system and compared it with foam generated using a single surfactant as well as with pure CO_2_ injection.

During oil production, the produced oil was collected in a glass bottle, which was weighed at regular intervals and analyzed for gas composition using a gas chromatography unit. After production, the mass and volume of the produced oil was used for material balance calculations to estimate fluid saturations, oil recovery factor, and CO_2_ storage factor. The key parameters used to assess the objectives of this study are explained as follows:

***Oil Recovery Factor (%)***: The oil recovery factor shows the percentage of oil that was recovered and ideally depicts the effectiveness of the injection technique to recover oil. RF was determined through material balance using the following formula:

(1) $$\text{RF}=\frac{V_{p}}{V_{o}}\cdot 100\text{\%}$$

where $V_{p}$ is the volume of produced oil, mL and $V_{o}$ is the volume of oil initially in the core samples, mL.

***Mobility Reduction Factor (MRF)***: This the ratio of the differential pressure with foam and without foam. This is used to evaluate the performance of foam flow in the porous media to control gas mobility. The formula is shown below:

(2) $$\text{MRF}=\frac{\Delta P_{f}}{\Delta P_{g}}=\frac{\frac{v\cdot \mu_{f}\cdot L}{k\cdot A}}{\frac{v\cdot \mu_{g}\cdot L}{k\cdot A}}$$

where $\Delta P_{f}$ and $\Delta P_{g}$ represent the differential pressure across the core samples for foam and gas respectively, MPa; *v* is the flow rate, ml/min; $\mu_{f}$ and $\mu_{g}$ represent the foam apparent viscosity and gas viscosity, respectively, cP; *L* is the length of the porous medium, m; *A* is the cross-sectional area of the porous medium, m^2^ and *k* is the permeability of the porous medium, mD.

The apparent viscosity of the foam used in this study at the thermobaric conditions has been earlier determined in our previous study [32]. The viscosity of CO_2_ at the thermobaric conditions was obtained from NIST [36].

***CO***_2_***Retention Factor (%)***: The effective CO_2_ retention factor is calculated by determining the volume of residual CO_2_ in the porous medium after the oil filtration process. By knowing the amount of CO_2_ injected from the pumps and using the gas chromatography unit installed at the producer to measure the volume of CO_2_ produced, we can determine the CO_2_ retention factor using the equation (3).

(3) $$\text{CO}{}_{2}\text{ Retention Factor}=\frac{V_{gi}-V_{gp}}{V_{gi}}\cdot 100\text{\%}$$

where $V_{gi}$ is the total volume of CO_2_ injected, mL; $V_{gp}$ is the total volume of CO_2_ produced, mL.

***CO***_2_***Theoretical Storage Capacity (g)***: This refers to the quantity of CO_2_ that can be potentially stored in a porous medium and it assumes that the pore spaces previously occupied by the produced fluids can be potentially utilized for CO_2_ storage. In our previous work, we have earlier defined these terms and can be calculated using the formula below:

(4) $$M_{t}=\rho_{\text{gas}}[RF\times \{Ah\phi (1-S_{wi})-V_{iw}+V_{pw}\}+C_{ws}\times \{Ah\phi S_{wi}+V_{iw}-V_{pw}\}+C_{os}\times \{Ah\phi (1-RF)+(1-S_{wi})\}]$$

where $M_{t}$ = theoretical CO_2_ storage capacity in the porous medium, kg; $\rho_{gas}$ = the density of CO_2_ in reservoir conditions, kg/$m^{3}$; *RF* = the recovery factor; *A* = the cross-sectional area of the porous medium, $m^{2}$; *h* = reservoir thickness, m; *ϕ* = porosity; $V_{iw}$ = the volume of injected water, $m^{3}$; $V_{pw}$ = the volume of produced water, $m^{3}$; $S_{wi}$ = irreducible water saturation; $C_{ws}$ = the solubility coefficient of CO_2_ in formation water; and $C_{os}$ = the solubility coefficient of CO_2_ in oil.

***CO***_2_***Storage Efficiency (%)***: Knowing the theoretical storage capacity, the CO_2_ storage efficiency can be calculated. Here, the CO_2_ storage efficiency is different from the effective retention factor which represents the actual CO_2_ retained in the porous media, presented earlier. This parameter rather refers to the efficiency of CO_2_ that can be stored in this particular reservoir, so it is predictive based on the theoretical storage capacity. Different studies have assessed ways to calculate this, however, in Bello et al. [32], we have included a modified formula that accounts for the CO_2_ loss due to surface and subsurface actions. We used a value of 5% for this [37], [38]. The formula is presented below:

(5) $${\text{CO}}_{2}\text{ storage efficiency}=\frac{{\text{CO}}_{2}\text{ injected}-({\text{CO}}_{2}\text{ produced}+{\text{CO}}_{2}\text{ loss})}{{\text{CO}}_{2}\text{ theoretical storage capacity}}$$

## 3 Results and discussion

The conventional core flooding procedure was conducted to evaluate the feasibility of injecting foams for CO_2_ utilization and storage, as well as to validate our previous hypotheses regarding CO_2_ foams generated with binary surfactant systems. A gas chromatography unit was installed at the outlet to monitor the gas composition dynamics at the producer. The porous media used in the experiments are replicate carbonate samples from the field, which mimic the high salinity conditions of the field and serve as potential media for geological CO_2_ storage.

**Figure 2:**
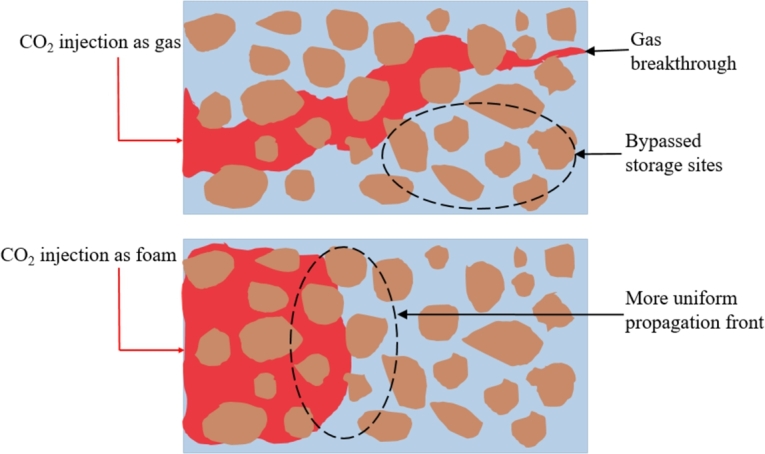
Storage in porous media when: (A) CO_2_ gas is injected and (B) CO_2_ foam is injected.

As illustrated in Fig. 2, our results showed that CO_2_ injection as foam, compared to gas, results in a more uniform propagation front and reduces gas breakthrough, thereby enhancing storage efficiency. All experiments were performed under the reservoir conditions of the field earlier described. This section details the various mechanisms involved in applying CO_2_ foams, not only as mobility control agents in subsurface porous media to ensure a more uniform propagation front but also as CO_2_ storage agents to increase CO_2_ storage by delaying gas breakthrough. The experimental results and their discussions are presented in the following subsections.

### 3.1 Mechanisms of foam for enhanced CO_2_ utilization and storage

CO_2_ mobility through porous media is hindered by its inherent low viscosity and high diffusivity [39], [15], [40]. Unlike resident fluids, the low viscosity of CO_2_ allows it to travel quickly, making it to fill up the porous media faster, which is economically beneficial to some extent. However, this rapid propagation makes CO_2_ less effective for displacing other fluids within the reservoir. A significant concern is the risk of viscous fingering, where CO_2_ forms narrow channels and spreads unevenly, creating pockets of unrecoverable oil [16]. This uncontrolled lateral spreading reduces the efficiency of CO_2_ storage and impacts the economic feasibility of such projects. In fluid injection processes within porous media, mass conservation requires that each unit volume of injected fluid displaces an equal volume of the existing reservoir fluid. For the process to be effective, the injected CO_2_ must displace a considerable amount of oil or brine while remaining well-distributed and retained within the reservoir.

**Figure 3:**
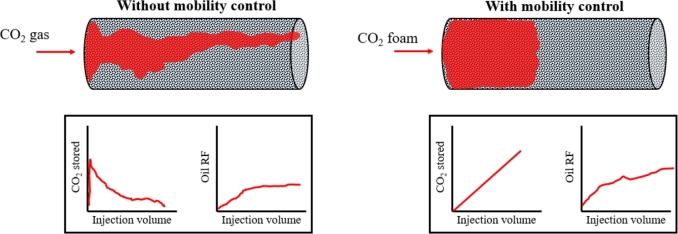
Observed trends during injection of CO_2_ as gas and foam in porous media.

Fig. 3 illustrates the impact of mobility control for CO_2_ utilization and storage. On the left side, we can see that as CO_2_ gas permeates the porous medium, the distribution is notably uneven, with the gas preferentially flowing through high-permeability channels and results in poor sweep efficiency, where large pore volumes are bypassed. Although, a substantial volume of oil might still be produced, it subsequently leads to an ineffective storage since majority of the gas injected is produced. In contrast, on the right side we see that foam spreads more uniformly across the medium, enhancing sweep efficiency by reducing the tendency of gas to breakthrough. This uniform distribution is indicative of improved mobility control, where the foam's increased viscosity helps to balance the displacement front [10]. Hence, as seen in the lower graphs in Fig. 3, a continuous increase in CO_2_ storage is expected since the pores from which oil is produced can effectively retain CO_2_ within the porous medium.

**Figure 4:**
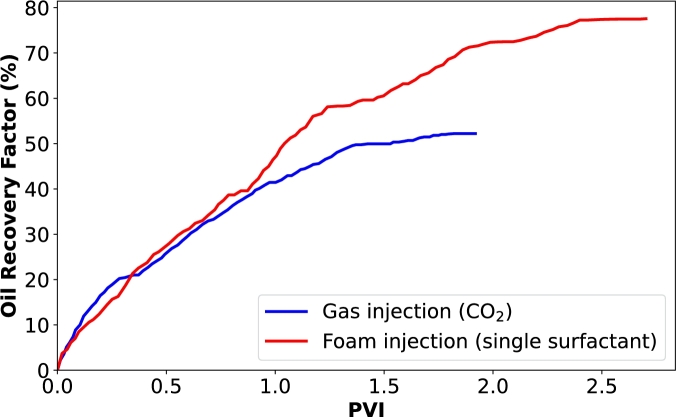
Oil recovery factor of two injection processes: CO_2_ injection as gas and CO_2_ injection as foam generated with a single surfactant (BETAINE).

**Figure 5:**
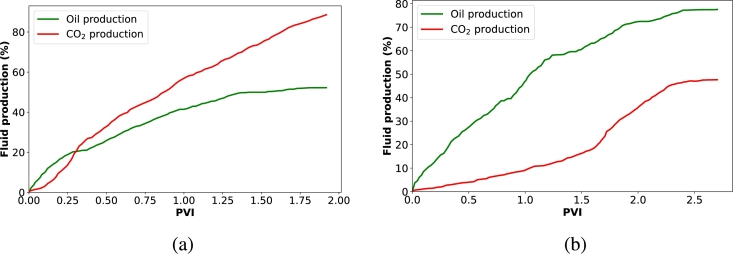
Oil and gas production factors of two injection processes: (a) CO_2_ injection as gas and (b) CO_2_ injection as foam generated with a single surfactant (BETAINE).

Fig. 4 presents the oil recovery results when CO_2_ was injected into the core samples as both gas and foam. Additionally, Fig. 5 illustrates the dynamics of oil and gas production at different pore volumes injected. In Fig. 4, we observe an improved oil recovery factor with foam injection, as previously explained and supported by various studies [10], [40], [14]. In Fig. 5a, we see that gas breakthrough occurred early at 0.3 PV during gas injection, while it was not evident during foam injection (Fig. 5b). This indicates that the gas quickly bypasses the oil and is produced at the outlet without sufficient storage during gas injection. However, this early breakthrough does not stop the oil production process, as there is a steady and continuous increase in cumulative oil production after 0.3 PV. This continuous oil production is because CO_2_ injection raises the reservoir pressure, providing an additional driving force for the oil to move towards the production well [41], [42], [43]. Additionally, oil production can continue through mechanisms such as the mass transfer of lighter components. CO_2_ can extract these lighter components from the oil, reducing its viscosity and allowing it to flow more freely towards the producer. Furthermore, the CO_2_-rich phase becomes more hydrocarbon-rich and can be produced together with CO_2_. However, the effectiveness of this process depends on the amount of CO_2_ that remains with the oil and the solubility coefficients of CO_2_ with the oil.

From Fig. 5a, it is evident that only around 20% of oil was displaced by CO_2_ before breakthrough, and more than 80% of the CO_2_ was produced after the flooding process, indicating the necessity of using a mobility control fluid for effective CO_2_ utilization and storage. When foam was injected, higher CO_2_ storage was achieved, which is significant for geological CO_2_ storage in depleted oil formations (Fig. 5b). Foams can clear void spaces of other residual fluids, making more space available for CO_2_ storage. Foam enhances the overall efficiency of the CO_2_ injection process in several key ways. Unlike CO_2_ gas, which can preferentially flow through high-permeability channels and leave behind unswept zones, foam exhibits a more uniform front [14], [25], [26]. The structure of foam creates a higher apparent viscosity, reducing the mobility of CO_2_ and forcing it to displace oil more effectively across the entire reservoir (Fig. 3). This results in a more uniform displacement of residual oil, ensuring that more void spaces within the reservoir are available for CO_2_ storage. Additionally, the ability of foam to block and divert the flow in higher permeability channels helps reduce the early breakthrough of CO_2_. This allows for greater contact time between CO_2_ and the reservoir rock and fluids, enhancing the mass transfer processes. The injection of foam also helps maintain pressure within the reservoir more effectively. By maintaining a higher and more consistent pressure, the foam ensures that CO_2_ remains in a supercritical state, which is denser and more efficient for storage purposes [24].

From Fig. 5, we can deduce that after the core flooding process, the percentage of CO_2_ retained for CO_2_ injection and foam injection are 11.40% and 52.35%, respectively. This significant increase in CO_2_ retention with foam injection can be attributed to the pore-plugging characteristics of the foam [26]. As mentioned earlier, gas trapping is a very significant pore level mechanism during foam flow in porous media. When foam bubbles plug pore throats, some bubbles become trapped while others are displaced by the injected surfactant solution. The surfactant solution bypasses the trapped foam bubbles by finding alternate flow channels. Once the flowing fraction of the foam is completely displaced by the surfactant solution, the flow channel for the foam phase becomes discontinuous. Consequently, the trapped foam fraction may remain permanently trapped due to this bypass mechanism. If the foam bubbles collapse during the injection process, it can degenerate and separate into gas and liquid, forming a continuous phase that advances through the porous medium and the encapsulated CO_2_ will be released as a free gas phase. However, the released CO_2_ will not be capable of miscibility with the residual oil due to its relatively lower pore pressure. Instead, it can only swell the oil, reduce its viscosity, and facilitate its production, or it may get stored in the porous medium. Thus, a possible mechanism of foams for CO_2_ storage in depleted oil formation is oil dissolution. Therefore, we assert that the following mechanisms are crucial during foam injection for the optimization of CO_2_ utilization and storage:

• Firstly, the co-injection of surfactant solution and CO_2_ generates foam, which increases flow resistance within the reservoir. This increased resistance forces CO_2_ to move through lower-permeability zones that would typically be bypassed during conventional CO_2_ injection, ensuring more areas of the reservoir are contacted by CO_2_. This leads to enhanced oil recovery and more efficient CO_2_ utilization.
• Secondly, the foaming agent significantly reduces the interfacial tension between crude oil and CO_2_. This reduction in IFT facilitates the mixing and dissolution of CO_2_ into the oil phase. Lowering the interfacial tension also increases the capillary number, helping to overcome capillary forces that typically trap oil within the porous media. Consequently, more oil is mobilized and displaced toward the production well. The presence of the foaming agent ensures a more thorough interaction between CO_2_ and oil, optimizing miscibility and, therefore, the efficiency of the oil recovery process. This not only promotes oil displacement but also enhances CO_2_ storage by increasing the contact area between CO_2_ and the rock surface, promoting better trapping of CO_2_ within the reservoir.
• More importantly, the Jamin effect aids foam in plugging the pore throats in the porous media. The foam bubbles create blockages in smaller pore spaces, effectively diverting CO_2_ flow into previously unswept or poorly swept zones. This increases sweep efficiency by expanding the volume of the reservoir contacted by the injected CO_2_. The Jamin effect is particularly beneficial in heterogeneous reservoirs with significant variations in permeability.

### 3.2 Contribution of binary surfactant systems to the improvement of CO_2_ utilization and storage

**Figure 6:**
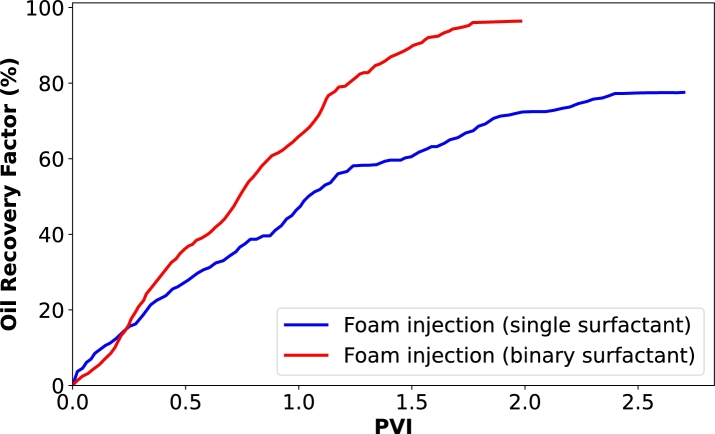
Oil recovery factor of two foam injection processes: foam generated with a single surfactant (BETAINE) and foam generated with a binary surfactant (BETFAR).

**Figure 7:**
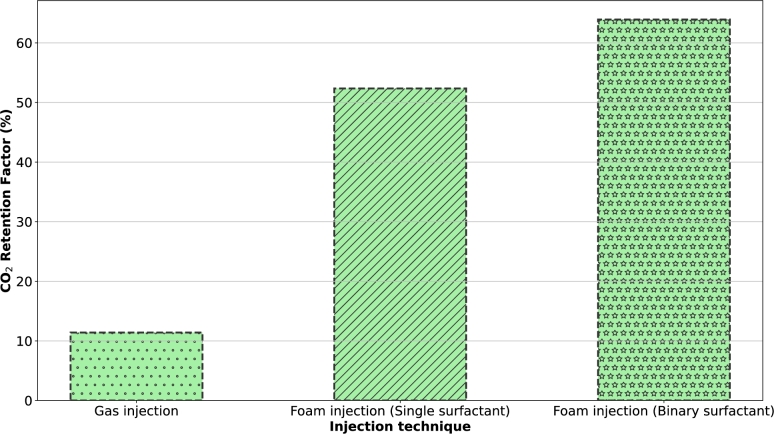
CO_2_ retention factor of the injection processes carried out in this study.

Figure 6, Figure 7 illustrate the improvement of CO_2_ utilization and storage through the application of binary surfactant systems as foaming agents as alternatives to single surfactants due to their beneficial synergistic effects. In our previous separate studies [32], [34], [33], based on our observations, we have made several hypotheses concerning this and the results in Figure 6, Figure 7 only confirm that by showing 24% and 22% increase in oil recovery and CO_2_ retention factor, respectively.

This could be due to various reasons which we have experimentally proven in our previous studies. Foams generated with binary surfactants are more stable and tolerant to harsh conditions known to destabilize foams such as high temperature, high salinity and oil presence. In Bello et al. [34], this was attributed to a precise tuning of the hydrophilic-lipophilic balance (HLB) between the surfactant molecules at the gas-liquid interface, where it was shown that the HLB value of the single surfactant (BETAINE) was 29.3 and was reduced to 15.9 when used in its binary form with BETFAR. This is change of HLB to a more lipophilic and less hydrophilic state means they have more affinity for the gas phase and would readily be more soluble in CO_2_. Thus, higher foamability and foam stability are expected.

**Figure 8:**
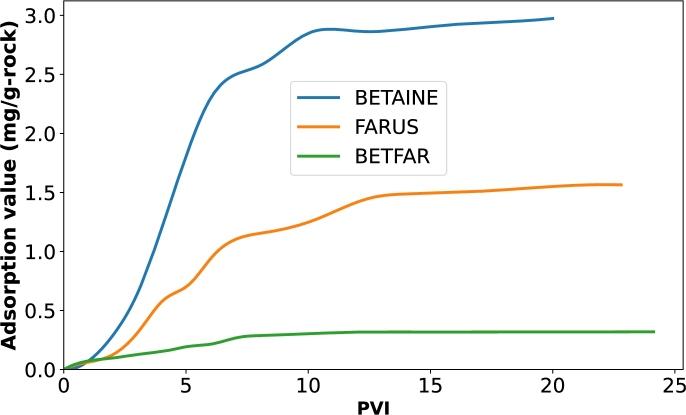
Dynamic adsorption curves for the single and binary surfactants systems conducted in a different study.

Furthermore, the adsorption of the foaming agent on rock surfaces play a great role in foam stability which in turn affects the extent of CO_2_ utilization and storage [44], [45]. This is because it is essential to keep as much as possible molecules of the foaming agent in the porous media to ensure sufficient foam generation. However, if the surfactant is lost to adsorption, there are less molecules available for foam generation, hence the foam becomes unstable and degenerates as soon as it get in contact with oil in the porous media. In Bello et al. [33], we have showed in a dynamic adsorption test how the binary surfactant system (BETFAR) showed an 89.26% reduction in rock adsorption compared to when just BETAINE was used (Fig. 8).

As explained earlier, the reasons were attributed to the increased solubility and decreased hydrophobicity of the surfactant system in the solution. As such, the binary surfactant molecules have more tendency to stay in the solution and avoid the interface where adsorption takes place. Furthermore, in Bello et al. [33], it was established that the binary surfactant systems undergo a monolayer mechanism of adsorption as opposed to the single surfactant. As such, once an adsorption was already formed, there was no further adsorption of the surfactant molecule. This allowed the utilization of the whole injected molecules to generate stable foam in the porous medium, whereas in the case of the single surfactant system, electrostatic interactions will keep occurring and surfactant adsorption will keep increasing, losing significant part of the injected surfactant molecules and causing the generation of unstable CO_2_ foam. Similar results were seen in AlSumaiti et al. [46], where the authors suggested that a reason for the no oil production which was noticed at the beginning of their flooding process was due to the fact that the initial surfactant injected was used up to satisfy rock adsorption.

### 3.3 Assessment of mobility control trends during CO_2_ utilization and storage

**Figure 9:**
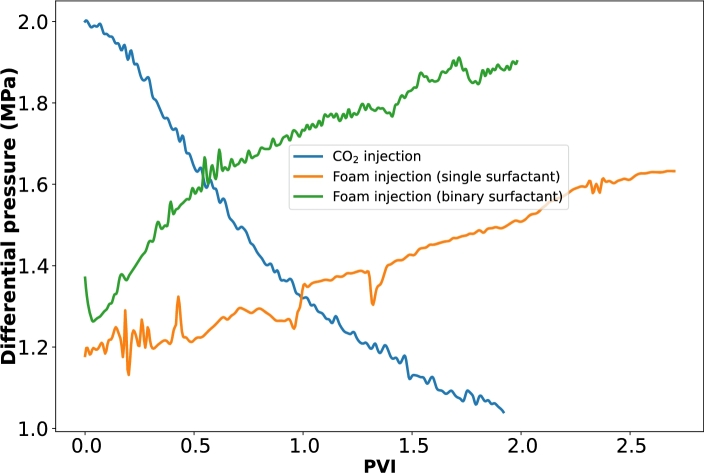
Differential pressure curves along the porous media.

Fig. 9 shows the variation in the differential pressure across the porous media during the filtration processes.

In Fig. 9, we see contrasting trends of differential pressure curves between the gas and foam injections. While the gas injection shows a decreasing trend, the foam injections show an increasing trend which confirms that the gas was being produced together with the oil during the CO_2_ injection. As such, the pressure drop in the porous medium kept decreasing. However, in the case of foam injection, with the increasing trend of the differential pressure drop, we can infer that foam displaces the oil and is replaced with CO_2_ or being retained in the porous medium, hence the differential pressure increases since more fluids are retained in the reservoir, even though there was a high oil recovery factor.

We believe that two mechanisms might have contributed to this increase in differential pressure. Firstly, the introduced foam possessed extremely high apparent viscosity and thus was able to propagate the oil front forward. Secondly, since foam can also act as a mobility control agent and can be used to divert flow to less permeable zones, the foam would prefer to enter into the large throats and then block them, accordingly, the relatively small pores and throats which could not be reached in the gas injection process due to the capillary force effect might have been swept by the CO_2_ foam.

We can also see the fluctuations in the differential pressure for the CO_2_ foam generated with single surfactant at the beginning of the process. This could be associated with the collapse and regeneration of the foam in the porous media. Compared to the injection of foam generated with the binary surfactant, the differential pressure increased relatively slowly and the fluctuation was even more pronounced, which is a clear indication of the inadequate foam generation. This poor performance could be due to the insufficient interaction between CO_2_ and the surfactant solution, a consequence of their mobility difference [47], [48]. It could also be due to high rock adsorption of the surfactant earlier depicted in Fig. 8, hence the remaining surfactant being insufficient for a stable foam generation.

**Figure 10:**
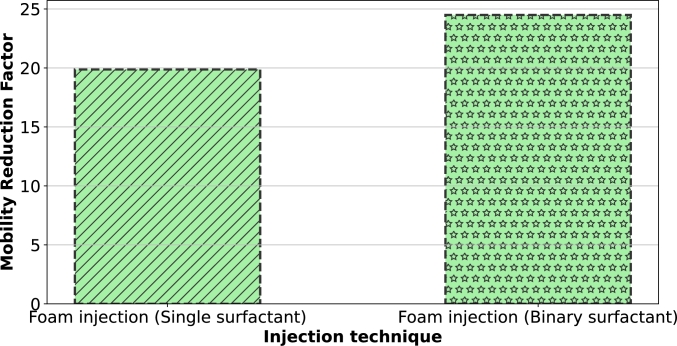
Mobility reduction factors of the 2 types of foam injected.

Fig. 10 shows mobility reduction factors for CO_2_ foam generated with single and binary surfactant systems. The mobility reduction factor was calculated as in equation (2). From Fig. 10, both mobility reduction factors were more than 1 which suggests that both foaming systems are effective mobility control agents. However, we can also notice a 23% increase in the mobility reduction factor for the foam generated with binary surfactant system which further explains why it is a better foaming agent and mobility control agent for CO_2_ utilization and storage.

**Figure 11:**
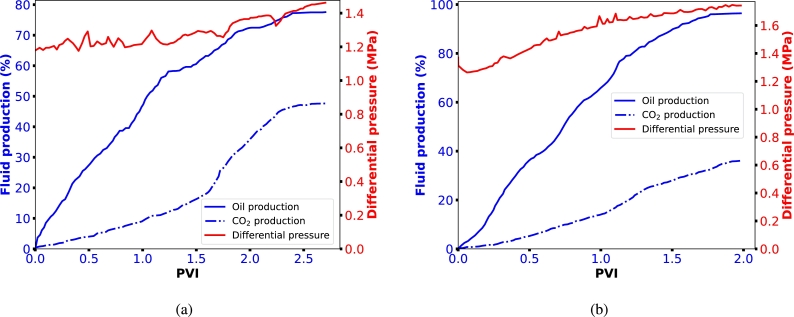
Differential pressure and fluid production curves (a) CO_2_ foam generated with single surfactant; (b) CO_2_ foam generated with binary surfactant.

According to the curves in Fig. 11, we can see that at the beginning of the foam flooding process, the oil recovery was quite high (>40%) with minimal production of CO_2_ gas, indicating that foam played an effective role in oil recovery at the beginning by acting as a mobility control agent. For the foam generated with single surfactant, this stage ended at 1.5 PV, and for foam generated with binary surfactant, the corresponding foam volume injected in this stage was around 1 PV. However, as the differential pressure began to increase, we begin to see significant volume of CO_2_ being produced, which was also found in Bachu and Adams [49]. Foam propagation was delayed with oil in the core, which can mainly be attributed to the fact that foam became less stable when contacting with oil. In Vassenden et al. [50], the transport of foam in the absence and presence of oil was investigated and the results showed that foam propagation was significantly delayed in the presence of oil. The volumes of foam generated with single surfactant which was injected before the increase of pressure and oil recovery were larger than in foam with binary surfactant. This result indicated that less CO_2_ foam existed in the core after injection. This could be due to 2 reasons. Firstly, the stability of foam generated with single surfactant in the presence of oil is poorer than foam generated with binary surfactant. Secondly, the solubilities of CO_2_ in both surfactant systems are totally different, and this also plays a significant role in stability.

We can also infer the mechanisms of foam stability in the presence of oil with the differential pressure curves. It is well known that foam would break down when it interacts with crude oil, although, the surfactant systems used in this study were designed and have been earlier investigated to have minimal deterioration in stability upon contact with crude oil. The differential pressure curves further indicate that oil destabilizes CO_2_ foam in porous media. The gradually increasing differential pressure can be interpreted as the effect of CO_2_ gas entrapment in pore throats. Without foam, the differential pressure will decrease rather than increase as in Fig. 9.

### 3.4 CO_2_ storage capacity and storage efficiency

The assessment parameters relating to CO_2_ storage were calculated as outline in section 2.2.3 for all the injection processes in this study and summarized as shown in Table 5.

**Table 5:** Assessment metrics for CO_2_ storage.

Injection process	Oil recovery factor	CO_2_ retention factor	Theoretical storage capacity	Storage efficiency
CO_2_ injection	52.22%	11.40%	1.468 g	17.26%
CO_2_ injection (single surfactant)	77.56%	52.35%	2.074 g	83.20%
CO_2_ injection (binary surfactant)	96.41%	63.91%	2.896 g	96.63%

It can be seen that the CO_2_ retention factors of the foam injection experiments were all between 52% and 63%, this further shows the efficiency of foam to improve CO_2_ storage, even in harsh conditions where CO_2_ solubility is low.

**Figure 12:**
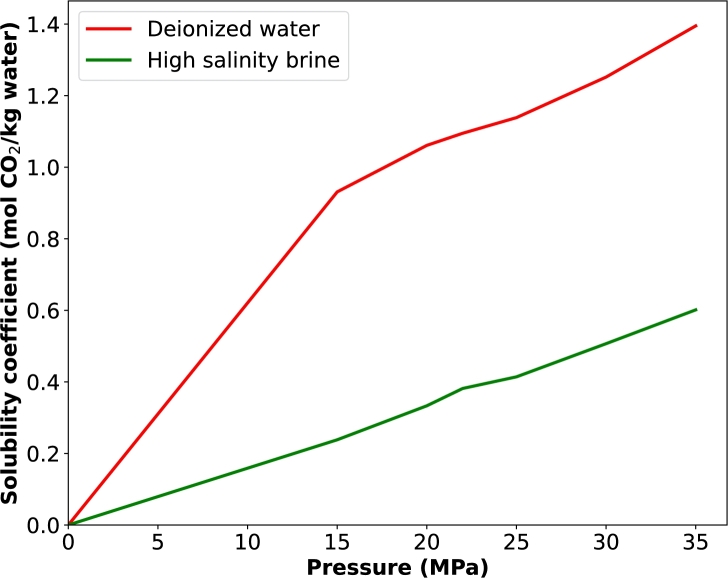
Solubility coefficients of CO_2_ in deionized water and in the high salinity brine used in this study (Data published in Penigin et al. [51]).

In Penigin et al. [51], we have shown the poor contribution of solubility trapping to CO_2_ storage in this field due to the high salinity of the brine. Thus, although CO_2_ is soluble in water, the increase in ions makes it difficult for CO_2_ solubility. As seen in Fig. 12, this is around 65% reduction in solubility. Therefore, we believe that residual trapping is also more dominant here, since the duration of the experiment is not sufficient enough for any significant mineral trapping. On the other hand, foam can enhance this process because the surfactants used are CO_2_ soluble and once CO_2_ dissolves in them, it is already in its aqueous phase and can easily in turn dissolve in the resident high salinity brine.

Consistent with the conclusion of the plugging effect, injecting pure CO_2_ is not conducive for CO_2_ sequestration, mainly because the gas channels and can breakthrough which makes the whole process ineffective. However, we see the highest storage efficiency is the case of foam injection because foams can delay the CO_2_ channeling time, increasing its swept volume and subsequently the storage capacity. These findings show the difference in the potential of the injectants to store and utilize CO_2_ in a depleted oil formation, as well as, position binary surfactant systems as potential alternatives to the traditional commercial surfactants for this purpose.

## 4 Conclusions

Given the role of CO_2_ in the global energy transition away from fossil fuels, it is essential to explore novel techniques of oil production that would also store significant volumes of CO_2_. This study investigates the use of foams as mobility control agents for subsurface CO_2_ utilization and storage. Three core flooding experiments were conducted using high salinity carbonate core samples: CO_2_ injection, CO_2_ foam generated with a single surfactant, and CO_2_ foam generated with a binary surfactant. Additionally, a gas chromatography unit was used to monitor the composition of the gas produced at the outlet at various intervals. The main conclusions are as follows:

• The generation of CO_2_ foam generated with binary surfactant system yielded the highest recovery factor of 96%, which is 24% higher than the foam generated with a single surfactant and 85% higher than the baseline CO_2_ injection experiment.
• Gas breakthrough occurred as early as 0.3 PV during CO_2_ injection, which was not evident during the foam injection processes.
• After the oil recovery process, CO_2_ gas injection retained only 11% of the injected CO_2_. In contrast, foam injections retained 52% and 64% of the injected CO_2_ for single and binary surfactant foam injection, respectively.
• Three mechanisms of foam for co-optimizing carbon utilization and storage were proposed: increased flow resistance, reduction of interfacial tension, and the Jamin effect.
• In depleted oil formations, the presence of oil can affect and degenerate the foam. However, the released gas will not be capable of mixing with the residual oil due to its relatively lower pore pressure, making further production unlikely.
• The foam generated with the binary surfactant system performed better due to its improved foamability, foam stability, and significantly lower rock adsorption compared to the foam generated with a single surfactant. This was further confirmed by a 23% increase in the mobility reduction factor.
• In the core samples used in this study, the theoretical storage capacities were determined to be 1.5 g, 2.1 g, and 2.9 g for CO_2_ injection, single surfactant foam injection, and binary surfactant foam injection, respectively.

## CRediT authorship contribution statement

**Ayomikun Bello:** Writing – review & editing, Writing – original draft, Visualization, Validation, Methodology, Investigation, Formal analysis, Data curation, Conceptualization. **Anastasia Ivanova:** Writing – review & editing, Supervision, Methodology, Investigation, Formal analysis. **Denis Bakulin:** Writing – review & editing, Visualization, Supervision, Resources, Methodology, Investigation, Formal analysis. **Kirill Maerle:** Writing – review & editing, Visualization, Investigation, Formal analysis, Data curation. **Denis Davydov:** Writing – review & editing, Visualization, Investigation, Formal analysis, Data curation. **Artem Penigin:** Writing – review & editing, Supervision, Resources, Project administration, Data curation. **Alexey Cheremisin:** Writing – review & editing, Visualization, Supervision, Resources, Project administration, Methodology, Funding acquisition, Data curation.

## Declaration of Competing Interest

The authors declare that they have no known competing financial interests or personal relationships that could have appeared to influence the work reported in this paper.

## Data Availability

The datasets used and/or analyzed during the current study are available from the corresponding author upon reasonable request.
